# PlotsOfData—A web app for visualizing data together with their summaries

**DOI:** 10.1371/journal.pbio.3000202

**Published:** 2019-03-27

**Authors:** Marten Postma, Joachim Goedhart

**Affiliations:** Swammerdam Institute for Life Sciences, Section of Molecular Cytology, van Leeuwenhoek Centre for Advanced Microscopy, University of Amsterdam, Amsterdam, the Netherlands

## Abstract

Reporting of the actual data in graphs and plots increases transparency and enables independent evaluation. On the other hand, data summaries are often used in graphs because they aid interpretation. To democratize state-of-the-art data visualization of raw data with a selection of statistical summaries, a freely available, open-source web app was written using R/shiny that uses the ggplot2 package for generating plots. Users can to choose how to display the data and which of the data summaries to add. In addition, the 95% confidence intervals (95CIs) can be added for visual inferences. By adjusting the visibility of the layers, the visualization of the raw data and their summaries can be tuned for optimal presentation and interpretation. The app is dubbed PlotsOfData and is available at https://huygens.science.uva.nl/PlotsOfData/.

## Abstract 2

This Community Page article describes PlotsOfData, an intuitive web-based tool for state-of-the-art visualization of data and statistics, without the need for coding skills.

## Introduction

Over the recent years, several groups have advocated the presentation of the actual data in graphs instead of data summaries [1–4]. Raw data can be visualized in different ways, including histograms and dot plots. Data summaries may be displayed to aid interpretation of the data. In addition, direct comparison of the different categories/conditions can be done by “visual inference” if 95% confidence intervals (95CIs) are supplied [5,6].

Several commercial software packages are available to draw data and their summaries. However, ideally, such tools should be open source, freely available, and allow contributions or modifications by users. One example of a free open-source web-based app to plot a combination of raw data and summaries is BoxPlotR (http://shiny.chemgrid.org/boxplotr/). The web-based app is described in a paper [7] that is remarkably well cited. Its popularity reflects a demand for easy-to-use applications that generate publication-quality data visualizations. However, this popular online tool is skewed towards box plots as data summaries and has hardly any options for customizing the combined display of data and summaries. Moreover, the plots are rather basic in appearance.

State-of-the art data visualization is possible with the R package ggplot2, which uses the ideas of a “grammar of graphics” to generate a graphic by using multiple layers of data [8]. The multilayered approach enables one to compose a graph from individual components, each of which can be independently adjusted. The option to apply transparency to the data layers adds to the flexibility. Yet, the high-quality data visualization provided by ggplot2 requires coding skills and understanding the concept of tidy data [9].

To democratize state-of-the-art data visualization of raw data with a selection of statistical summaries, we generated a web tool that we dubbed PlotsOfData. The web tool uses ggplot2 to compose the graphs and handles data in ordinary spreadsheet (wide) format as well as the tidy data format. Because creating graphs with PlotsOfData does not require coding skills, the high-quality data visualization provided by ggplot2 is now available to anyone. Some of the features of PlotsOfData will be highlighted below.

## Availability and code

PlotsOfData is available online at: https://huygens.science.uva.nl/PlotsOfData/. The app uses the shiny package and was written in R, using R (https://www.r-project.org) and Rstudio (https://www.rstudio.com). It uses several freely available packages (shiny, ggplot2, dplyr, tidyr, readr, magrittr, ggbeeswarm, readxl, DT). The source code of the current version (v1.0.5) is archived at zenodo: https://doi.org/10.5281/zenodo.2582567. An up-to-date version is available at Github together with information on how to install and run the app locally: https://github.com/JoachimGoedhart/PlotsOfData.

When the PlotsOfData R/shiny script is downloaded from Github, the web app can be started from R or Rstudio and used offline.

## Issues and updates

The Github page of PlotsOfData can be used to trace changes between different versions. The page has a list with issues and feature requests (https://github.com/JoachimGoedhart/PlotsOfData/issues) that (i) is used to communicate issues in a clear way and (ii) provides a way to invite people to contribute and help to improve the code. Users of PlotsOfData can report an issue or request a feature on the Github page, or they can contact the developers by email or Twitter. Up-to-date contact information is found on the “About” page of the app.

## Data input and structure

**Fig 1:**
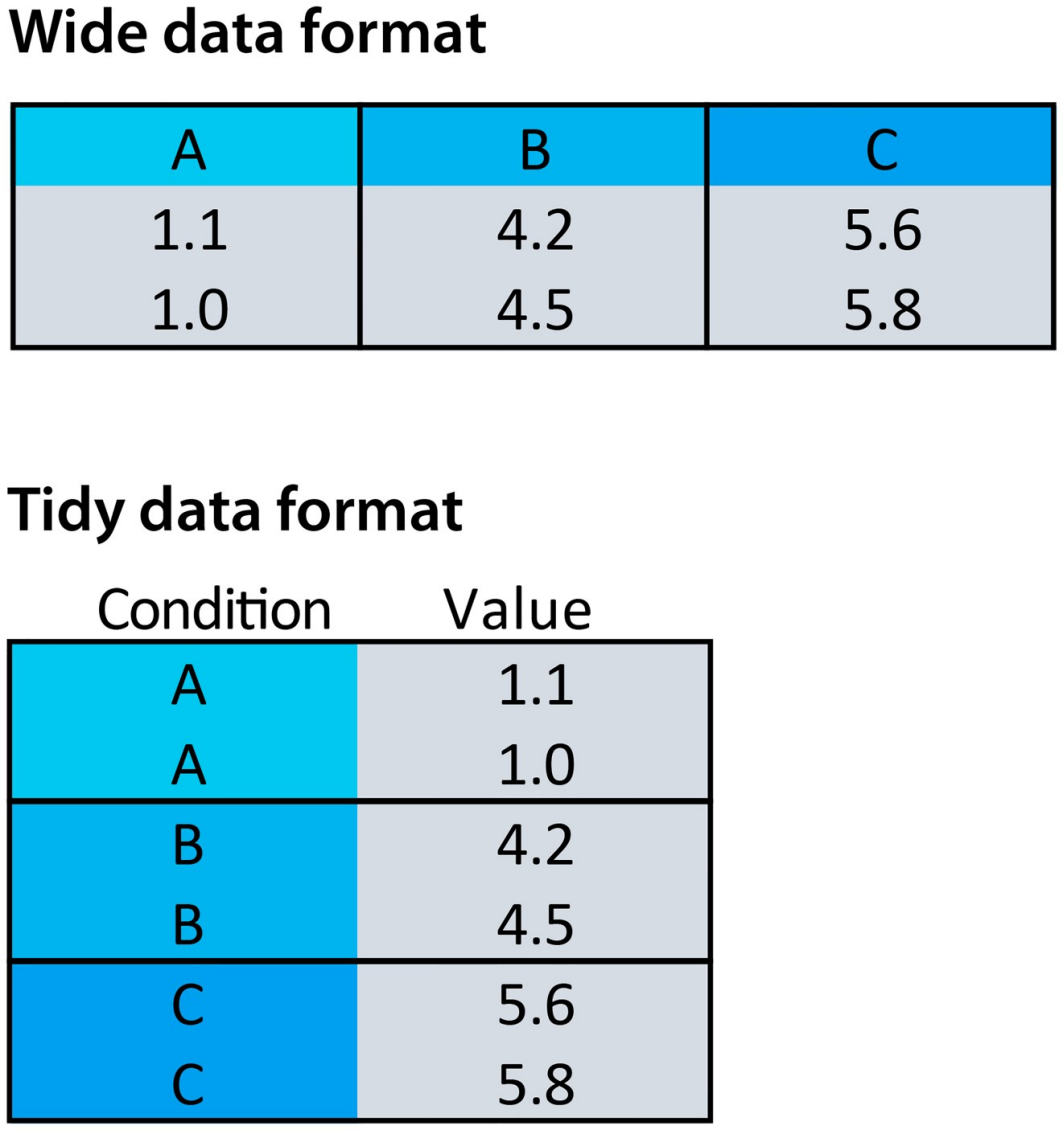
The wide versus tidy data format. In the wide (spreadsheet-like) data format each column represents a condition. In the tidy format each column is a single variable and each row is an observation.

The data can be provided by copying and pasting into a text box or by upload of two file formats, i.e., comma-separated values (CSV) or XLS(X) (Excel) format. Several delimiters (comma, tab, semicolon, space) are recognized. Two example files are available in the app for testing the application. These files are also available as CSV files (https://github.com/JoachimGoedhart/PlotsOfData). Data may exist in different formats [10]. The native structure that ggplot2 uses is the “tidy” format [9], and this data structure is accepted as input. However, (raw) data are often stored in a wide, spreadsheet-type structure in which each column reflects a condition. The different structures of the wide format and tidy format are illustrated in Fig 1. This visual explanation is also available in the “Data upload” tab of the app. The wide format is the default data structure that is used by the app. Users may select columns from the spreadsheet data that should not be included in the graph. After the input, the wide data are converted into tidy format, assuming that each column is a condition with a single row header that lists each condition. To promote the understanding of tidy data, the data can be downloaded in a tidy format. The defaults of the data input can be changed by adding input to the HTML address. The ?data = [1/2/3/4];[T] tag can be used to change the input and structure. The value between the brackets lists a choice for a parameter. For instance, the HTML address below starts PlotsOfData with Data upload set to option 2 (“Example 2”) and tidy set to T(TRUE) for the input: https://huygens.science.uva.nl/PlotsOfData/?data=2;T.

These instructions can also be found under the “About” tab, with a complete list of available input variables.

## Data visibility

The data are shown as transparent dots. Offset can be added to the dots to avoids overlap for a larger number of dots. When quasirandom is selected as the offset, the dots are shown according to the data distribution (i.e., similar to a violin plot). Both the offset and user-defined visibility of the raw data can be adjusted to optimize the visualization of the raw data. For low numbers of data, it is pertinent to plot the data [1,3,11]. For very large numbers of data, i.e., when the dots show substantial overlap, one may consider to make the data fully transparent and only plot their distribution with a violin plot.

## Statistical summary

Any of four statistics can be added to summarize the data, i.e., median, mean, box plot, or violin plot. The median is not sensitive to outliers and as such is a robust indicator of the central value [12]. The median is also indicated in the box plot [13] and violin plot by a horizontal line. Because both box plots and violin plots reflect data distribution, they are only appropriate if sufficient data are provided (the lower limit is now set at *n* = 10).

To enable inference by eye, the 95CI can be added to the plot [5,6]. For box plots, the 95CI is indicated by notches [13]. The original definition of notches was reported by McGill and colleagues, but their calculation does not correct for small sample size [13]. Therefore, notched box plots should be used with care for smaller samples (*n* < 20).

The 95CI that is calculated when the median or violin plot is selected is calculated by bootstrapping (1,000 samples) and determining the 95CI from the 2.5th and 97.5th percentile [14]. Because bootstrapping requires a representative sample from the population, it is only suitable if sufficient data are present. Because the underlying population that was sampled from is unknown, it is, per definition, unclear what “sufficient” means. To reduce the chances that the 95CI does not correctly reflect the population, we have set the minimum number of data points in the app at 10 per condition for the calculation of the 95CI. Users who want to add box plots, violin plots, or 95CI will be notified by a pop-up window that this is not possible due to small sample size.

**Fig 2:**
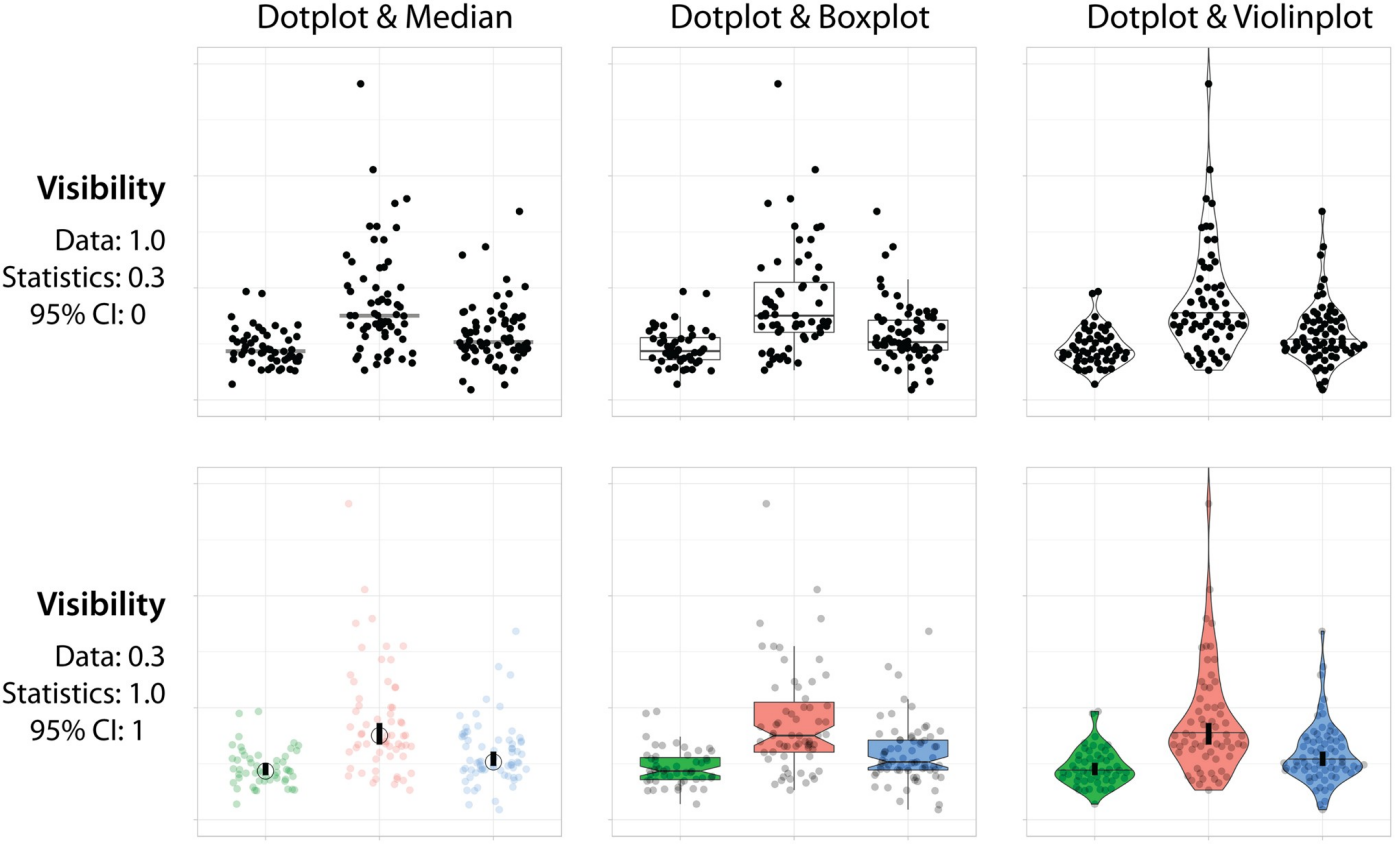
An overview of data visualizations generated using PlotsOfData. The sample data that are available in PlotsOfData are presented together with statistics in different ways. Upper row, a data-centered presentation, with prominent display of the data as dot plots and the statistics displayed with a visibility of 0.3. Lower row, a statistics-centered presentation that shows the data at lower visibility (0.3) and prominently shows the statistics and the 95CIs. Horizontal lines (or a circle in the lower left panel) display the median value, and vertical bars show the 95CI. For box plots the 95CI is indicated by notches. 95CI, 95% confidence interval.

Fig 2 demonstrates the use of the median, a box plot, or a violin plot as a summary. The box plot and violin plot also convey information on the distribution of the data. In the lower row, the 95CI is depicted to make visual inferences. When the 95CIs of two independent conditions do not overlap, this is a strong indicator of a statistical difference between these conditions [5]. The difference can be quantified by calculating the effect size.

## Transparent layers

The raw data can be combined with any of four data summaries, i.e., mean, median, box plot, and violin plot. In addition, the 95CI can be added for inferences. The simultaneous visualization of the data and statistics is achieved by using (transparent) layers. For optimal visualization, the order of the layers is defined as follows (from first to last, with last appearing on top): (1) box- or violin plot, (2) raw data, (3) mean or median, and (4) 95CI. The visualization can be optimized by user-defined visibility of the layers. Fig 2 demonstrates how the “visibility” of the data and statistics can be adjusted for a visualization that focusses on the data (upper row) or one that stresses the statistics (lower row). A description of how these figures can be generated with the app can be found in a supplemental note (see S1 Text).

## Ordering

The conditions can be sorted in three different ways. First, the conditions can be visualized in alphabetical order, which is the default order for ggplot2. Second, the categories can be shown in the same order as provided (by copy and paste or in the uploaded file). Third, the conditions can be sorted according to the median value.

## Table with statistics

The statistics that are selected for visualizing the data are also documented in a table on a separate tab. The calculated statistics include the mean, standard deviation (SD), standard error of the mean (SEM), 95CI of the mean, median, median absolute deviation (MAD), interquartile range (IQR), quartile 1 (Q1), quartile 3 (Q3), and the 95CI of the median. The mean and median are measures of centrality and reflect the typical value of a distribution. The SD, IQR, and MAD are measures of dispersion that indicate the variability of a distribution. The SEM and 95CI are inferential statistics that are used to infer information about the population distribution that the sample was taken from.

**Fig 3:**
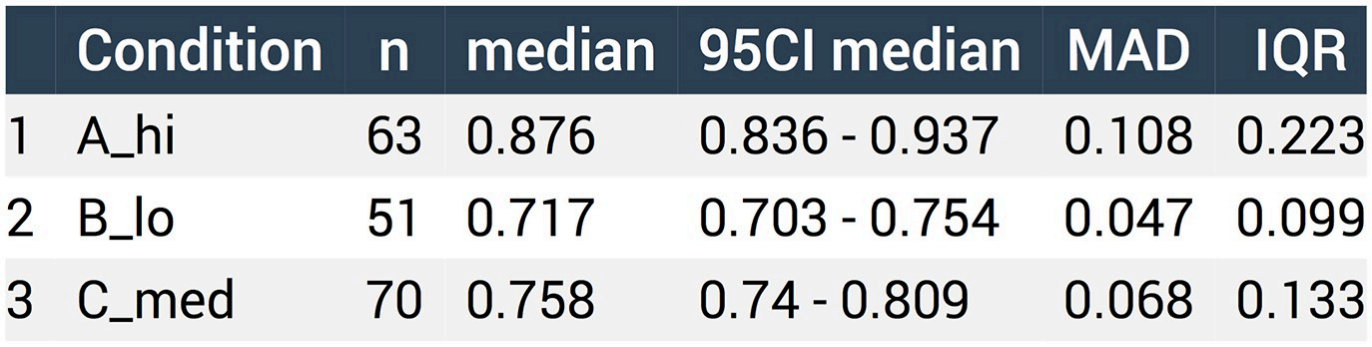
The statistical summary of the data can be downloaded in several different formats. Here, the outputs for the data related to Fig 2 when downloaded as PDF are shown. IQR, interquartile range; MAD, median absolute deviation; 95CI, 95% confidence interval.

The default statistics listed in the table depend on the summary statistics that are shown in the graph. For instance, when the mean is selected the mean and SD are shown in the table, but when the median is selected the median and the MAD are included. The user can change the default statistics that are shown in the table and rearrange their order by drag-and-dropping the columns. Moreover, the number of digits that is shown can be adjusted. The table can be exported in CSV or XLSX format, to PDF or copied to the clipboard. Fig 3 shows an output example of the statistical summary related to the data that are shown in Fig 2.

## Plot layout

To further optimize the data visualization, several options to change the layout of the plot have been implemented. The plot can be rotated 90 degrees, which generally improves readability of the labels for the conditions. The grid can be removed. The scale can be adjusted, and there is an option for a log10 scale.

In the default output, no colors are used. Color can be added to the data, the statistics, or both. There is an option to use the standard palette, or several color-blind safe palettes that are optimized for categorical, qualitative data (https://personal.sron.nl/~pault/). Finally, user-defined colors can be added by using color names or hexadecimal codes. The user-defined colors are added to the conditions in the alphabetical order of the conditions, even if the conditions are shown in a different order.

**Fig 4:**
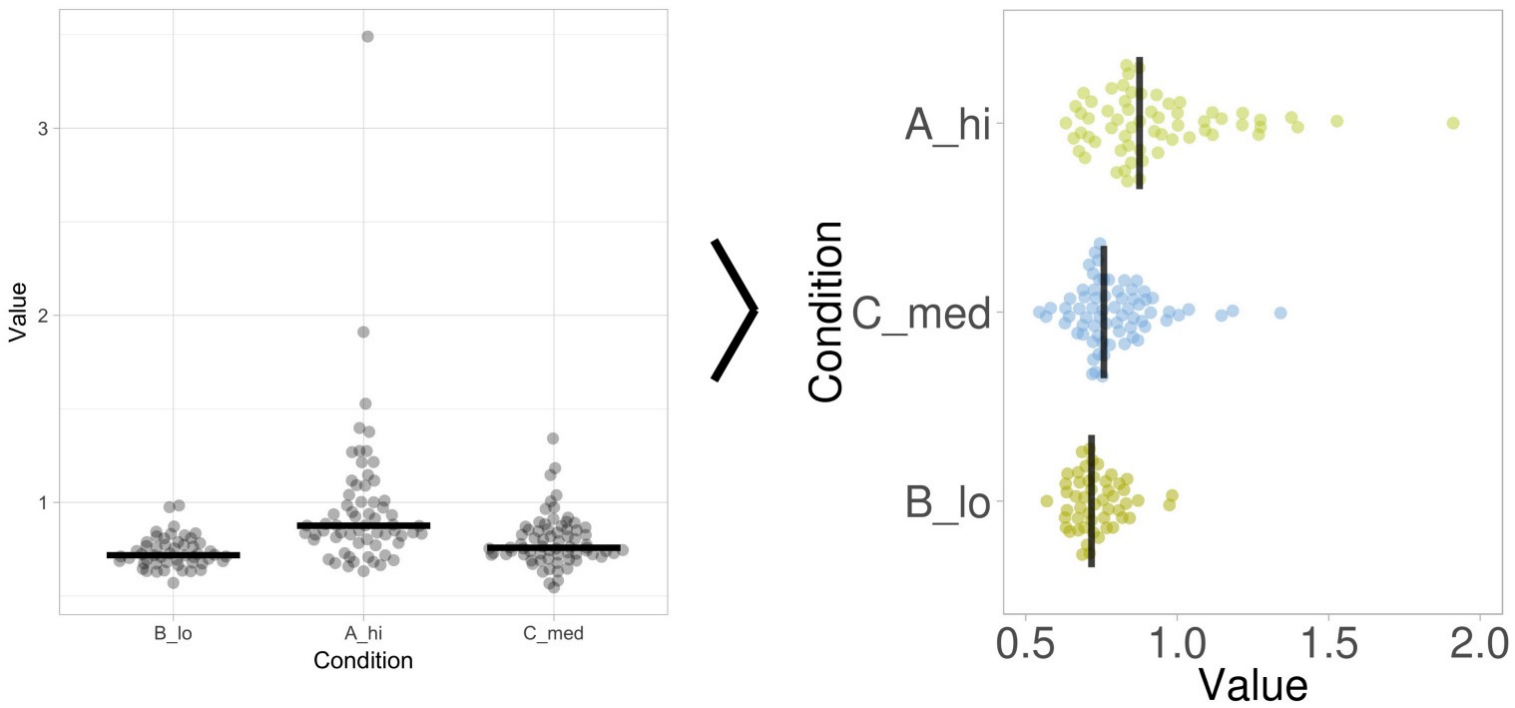
The layout of the graph generated by PlotsOfData can be further refined. The standard output of PlotsOfData (left panel) can be tuned to enhance the presentation. The graph on the right is generated by changing the visibility of the statistics, sorting of the conditions according to median value, rotating the plot, removing the grid, adjusting the scale, adding colors for the data, and adding a figure description.

The labels for the axes and the plot title can be changed. The size of the plot and the font size can be modified. Finally, there is an option to add a figure caption. The caption responds dynamically when the plot is changed. An example of how the standard output can be modified is shown in Fig 4.

The settings that are selected to generate a plot can be “cloned.” This action will generate a HTML address that can be bookmarked. The customized HTML link will launch PlotsOfData with the user-specified settings. If the plot was generated with CSV data from a URL, the plot is automatically presented. In the case that the plot was made by adding the data with copy and paste or uploading a local file, the data need to be provided after launching PlotsOfData from the HTML address. S1 Text has more information on the parameters that can be passed through the HTML link.

## Output

The graphs can be directly saved from the web browser that is running the app (e.g., by drag-and-drop from the web browser). Alternatively, two options are available for downloading the figure, PDF and PNG. The PNG format is lossless and can be readily converted to other bitmap-type formats that are suitable for presentation or incorporation into (multi-panel) figures. The PDF format is vector based and can be imported into any software package that handles vector-based graphics for further adjustment of the layout. The optional figure description can be copied via the clipboard to a text editor. Alternatively, it can be included with the plot by making a screenshot.

The table with the summary can be copied to the clipboard and exported in several formats, including PDF, XLSX, or CSV.

## Conclusion

PlotsOfData was generated with the aim to enable anyone to visualize their data in combination with a selection of summaries. The user-defined mixing of dot plots with statistical summaries should improve the creation of graphs and visual inferences. The use of box plots, violin plots, and 95CI requires sufficient data. It is, however, not agreed upon what “sufficient” implies. In the app, the minimum is set at *n* = 10 for showing box plots, violin plots, and 95%CI, but it is up to the user to critically assess whether this is sufficient. The source code for the app is available and the threshold can be readily changed in the code. Regardless of the statistics that are shown, it is recommended to plot the data for low to medium *n* [1,3,11,12].

Finally, we hope that the high-quality plots created with PlotsOfData will improve transparent communication of scientific data, which will be beneficial for both researchers and their audience.

## Supporting information

S1 Text Passing parameters to PlotsOfData through the HTML address. (DOC)

## Abbreviations

95CI: 95% confidence interval
CSV: comma-separated value
IQR: interquartile range
MAD: median absolute deviation
Q1: quartile 1
Q3: quartile 3
SD: standard deviation
SEM: standard error of the mean
