# Supplementary material for: PlotsOfData—A web app for visualizing data together with their summaries
Source: PLoS Biol. 2019 Mar 27;17(3):e3000202. doi: 10.1371/journal.pbio.3000202 (PMC6453475; doi:10.1371/journal.pbio.3000202)
Supplement: S1 Text — (DOC) [file pbio.3000202.s001.doc]

**S1 Text – Passing parameters to PlotsOfData through the HTML address**

**Reproducing figure 2 and 3**

The plots of figure 2 and 3 can be reproduced by adding a ‘query’ to the HTML address. The full HTML address for each of the figures is listed below. An explanation of generating presets and the possible parameters that can be passed through the query can be found below.

Figure 2; Upper row from left to right:

<https://huygens.science.uva.nl/PlotsOfData/?vis=random;1;median;;0.3;none&layout=;;T;;0.5,2;;;;>

<https://huygens.science.uva.nl/PlotsOfData/?vis=random;1;box;;0.3;none&layout=;;T;;0.5,2;;;;>

<https://huygens.science.uva.nl/PlotsOfData/?vis=quasirandom;1;violin;;0.3;none&layout=;;T;;0.5,2;;;;>

Figure 2; Lower row from left to right:

<https://huygens.science.uva.nl/PlotsOfData/?vis=random;0.3;median;TRUE;1;none&layout=;;T;;0.5,2;TRUE;;;>

<https://huygens.science.uva.nl/PlotsOfData/?vis=random;0.3;box;TRUE;1;none&layout=;;T;;0.5,2;;TRUE;;>

<https://huygens.science.uva.nl/PlotsOfData/?vis=quasirandom;0.3;violin;TRUE;1;none&layout=;;T;;0.5,2;;TRUE;;>

Figure 4; right:

[https://huygens.science.uva.nl:/PlotsOfData/?data=1;;none;none&vis=quasirandom;0.5;median;;0.5;median&layout=TRUE;TRUE;TRUE;;0.5,2;TRUE;;4;TRUE;480;480&color=Condition;none&label=;;;;;T;28;28;TRUE&](https://huygens.science.uva.nl/PlotsOfData/?data=1;;none;none&vis=quasirandom;0.5;median;;0.5;median&layout=TRUE;TRUE;TRUE;;0.5,2;TRUE;;4;TRUE;480;480&color=Condition;none&label=;;;;;T;28;28;TRUE&)

**Application**

This method for setting a specific layout is useful for defining presets. For instance, when you often want to plot rotated violinplots without grid and with a 95CI you could bookmark:

<https://huygens.science.uva.nl/PlotsOfData/?vis=quasirandom;0.5;violin;TRUE;1;none&layout=T;T>

The method is even more effective when the data (in CSV format) is available from a URL. For instance, a dataset from zenodo (https://zenodo.org/record/2545922/files/FRET-efficiency_mTq2.csv) can be used as input and automatically plotted with previously defined parameters:

https://huygens.science.uva.nl:/PlotsOfData/?data=5;;none;none&vis=random;0.3;median;;1;median&layout=TRUE;TRUE;;;;TRUE;;5;;480;480&color=Condition;green,green,red,orange,green,orange,red,red,red&label=TRUE;mTurquoise2 paired with different FPs;TRUE;Acceptor;FRET efficiency [%];TRUE&url=https://zenodo.org/record/2545922/files/FRET-efficiency_mTq2.csv

The address is rather lengthy due to all the parameters that are necessary, but these can be ‘hidden’ by adding the hyperlink to text, for instance [this link](https://huygens.science.uva.nl/PlotsOfData/?data=5;;none;none&vis=random;0.3;median;;1;median&layout=TRUE;TRUE;;;;TRUE;;5;;480;480&color=Condition;green,green,red,orange,green,orange,red,red,red&label=TRUE;mTurquoise2 paired with different FPs;TRUE;Acceptor;FRET efficiency %5B%25%5D;TRUE&url=https://zenodo.org/record/2545922/files/FRET-efficiency_mTq2.csv).

**Generating presets**

Users can generate the HTML address with the presets by using the ‘clone current setting’ button. This will generate the HTML code which can be copied for later use. Again, this is most effective when a URL is specified since it will automatically generate a plot when the HTML address is accessed.

When no URL is available, the user needs to upload the data after launching PlotsOfData from the HTML address.

**Background information on setting parameters through the HTML address**

Passing parameters through the HTML address can be used to define a visualization and alter the standard layout. There are several queries (?data, ?vis, ..) that can be used. Each of the queries can hold multiple parameters. The queries are separated by an ampersand (&) and the parameters are separated by a semicolon(;).

Parameters that need no change (i.e. remain default) should be left empty. The parameters are defined by their position so their order is important. Below, the parameters than can be changed through the HTML address are indicated, with the options between brackets:

?data

[1] data_input {1/2/3/4/5}

[2] tidyInput {T}

?vis

[1] jitter_type {quasirandom/random/stripes/none}

[2] alphaInput {0-1}

[3] summaryInput {median/mean/box/violin}

[4] add_CI {T}

[5] alphaInput_summ {0-1}

[6] ordered {none/median/alphabet}

?layout

[1] rotate_plot {T}

[2] no_grid {T}

[3] change_scale {T}

[4] scale_log_10 {T}

[5] range -> number (minimum value),number (maximum value)

[6] color_data {T}

[7] color_stats {T}

[8] adjustcolors {1/2/3/4/5}

[9] add_description {T}

[10] plot_height -> number reflecting pixels

[11] plot_width -> number reflecting pixels

?color

[1] colour_list {Condition}

[2] user_color_list -> list of colors separated by comma’s

?label

[1] add_title {T}

[2] title -> text

[3] label_axes {T}

[4] lab_x -> text

[5] lab_y -> text

[6] adj_fnt_sz {T}

[7] fnt_sz_ttl -> number

[8] fnt_sz_ax -> number

[9] add_description {T}

?url

query[[‘url’]] URL -> URL
